# Supplementary material for: Maternal Diet, Nutritional Status, and Birth-Related Factors Influencing Offspring’s Bone Mineral Density: A Narrative Review of Observational, Cohort, and Randomized Controlled Trials
Source: Nutrients. 2021 Jul 4;13(7):2302. doi: 10.3390/nu13072302 (PMC8308284; doi:10.3390/nu13072302)
Supplement: Supplementary file 1 [file nutrients-13-02302-s001.zip › nutrients-1239780-supplementary.pdf]

nutrients

MDPI

Review

Maternal Diet, Nutritional Status, and Birth-Related Factors Influencing Offspring's Bone Mineral Density: A Narrative Review of Observational, Cohort, and Randomized Controlled Trials

Daria Masztalerz-Kozubek, Monika A. Zielinska-Pukos and Jadwiga Hamulka*

Table S1. Detailed data about dietary assessment method, maternal dietary intake in pregnancy and type of data used for statistical analysis.

Country, Year [Reference]	Maternal Dietary Intake Assessment (gestation)	Detailed Information about Dietary Assessment Method	Reported Maternal Dietary Intake	Data Used for Analysis
Netherlands, 2013 [19]	FFQ (1st trimester)	Modified version of the validated semiquantitative FFQ by Klipstein-Grobusch, after validation [90]<br><br>293 food items in the previous 3 months	Energy 2152 ± 504 kcal<br>protein 78.5 ± 11.7 g/d; carbohydrate 236 ± 30 g/d; fat 73.6 ± 10.7 g/d;<br>calcium 1108 ± 311 mg/d; phosphorus 1443 ± 241 mg/d; magnesium 339 ± 56 mg/d	Quintiles of energy from macronutrients and mineral intake
UK, 2005 [32]	FFQ (32 wk)	Semiquantitative FFQ based on previous FFQ used in UK (without validation after modifications) [91]<br><br>75 food items in the previous 3 months	Energy 7505 ± 1967 kJ<br>protein 67.7 ± 18 g/d; carbohydrate 223 ± 61.5 g/d; fiber 20.3 ± 6.31 g/d; starch 121 ± 36 g/d; sugar 102 ± 38 g/d; intrinsic/milk sugar 41.6 ± 12 g/d; extrinsic non-milk 60.6 ± 34 g/d; total fat 70.6 ± 22.7 g/d; saturates 28.9 ± 10.9 g/d; monounsaturates 24.8 ± 8.15 g/d; polyunsaturates 12.7 ± 5.75 g/d; omega-3 0.155 ± 0.146 g/d<br>calcium 952 ± 275 mg/d; phosphorus 1339 ± 338 mg/d; magnesium 261 ± 74.2 mg/d; potassium 2626 ± 636 mg/d; sodium 2239 ± 619 mg/d; total zinc 8.55 ± 2.31 mg/d; iron 10.8 ± 3.15 mg/d; retinol 490 ± 328 µg/d; riboflavin 1.73 ± 0.513 mg/d; carotene 2291 ± 1164 µg/d; folate 257 ± 69.6 µg/d; thiamin 1.46 ± 0.401 mg/d; niacin 16.3 ± 4.86 mg/d; vitamin B_6 1.87 ± 0.502 mg/d; vitamin C 84.3 ± 33.8 mg/d; vitamin D 3.11 ± 1.45 µg/d; vitamin E 8.57 ± 4.35 mg/d	Quantitative data
Australia, 2000 [20]	FFQ (after birth)	Self-administered semiquantitative FFQ<br><br>179 food items in the 3rd pregnancy trimester	Energy 15398 ± 8675 kJ<br>protein 155 ± 94 g/d; carbohydrate 417 ± 231 g/d; fat 162 ± 101 g/d<br>calcium 1905 ± 1310 mg/d; phosphorus 2767 ± 1655 mg/d; magnesium 504 ± 280 mg/d, potassium 3084 ± 1042 mg/d	Tertiles of density of selected nutrients

Australia, 2010 [21]	FFQ (3rd trimester)	Self-administered semiquantitative FFQ<br><br>179 food items in the 3rd pregnancy trimester	Energy 13264 ± 4599 kJ; fat 162 ± 101 g/d<br>protein 128 ± 45 g/d; carbohydrate 375 ± 139 g/d<br>calcium 1677 ± 841 mg/d; phosphorus 2314 ± 898 mg/d; magnesium 429 ± 149 mg/d	Quantitative data (density of selected nutrients)
UK, 2009 [33]	FFQ (15, 32 wk)	Validated semiquantitative FFQ administered by nurse [92]<br><br>100 food items in the previous 3 months	PDS in early pregnancy 0.14 ± 2.2; PDS in late pregnancy 0.26 ± 2.1<br>protein 84.7 (73 - 99.1) g/d<br>calcium 1230.1 (1019.6 - 1502.3) mg/d	Quantitative data (PDS)
India, 2006 [24]	FFQ and 24-h recall (18, 28 wk)	Developed for this community and validated FFQ and 24-h recall [93]<br><br>111 items in the previous 3 months	Frequency of intake per month:<br>milk 18 and 28 wk 6 (0 – 16); milk products 18 wk 61 (36 – 66), 28 wk 61 (34 – 66); pulses 18 wk 52 (35 – 73), 28 wk 47 (31 – 69); fish, meat or eggs 18 wk 2 (0 – 8), 28 wk 1 (0 – 8); green leafy vegetables 18 wk 10 (5 – 21), 28 wk 6 (2 – 14); fruit 18 wk 28 (14 – 47), 28 wk 20 (10 – 37); all calcium-rich 18 wk 145 (109 – 182), 28 wk 134 (103 – 169)<br>calcium 18 wk 274 (223 – 354), 28 wk 268 (208 – 332) mg/d; phosphorus 18 wk 1033 (911 – 1120), 28 wk 1016 (890 – 1174) mg/d; magnesium 18 wk 477 (416 – 548), 28 wk 477 (416 – 545) mg/d	Quantitative data (frequency of intake)
UK, 2001 [31]	FFQ (in early and late pregnancy)	Validated semiquantitative FFQ administered by nurse [92]<br><br>100 food items in the previous 3 months	Energy 14.7 wk 2293 (1868 – 2862) kcal/d, 28 wk 2551 (2140 – 3121) kcal/d<br>green vegetable intake 14.7 wk 6.7 (4.4 – 10.0) portions/wk, 28 wk 6.2 (3.5 – 9.0) portions/wk<br>calcium 14.7 wk 1183 (983 – 1554) mg/d, 28 wk 1320 (1049 – 1665) mg/d	Quantitative data after log-transformation (calcium and green leafy vegetables)
Australia, 2017 [30]	FFQ (28-32 wk)	Validated Victorian Cancer Council FFQ [94]<br><br>100 food items in the previous 12 months	Energy 7831 (6506 – 9327) kJ/d<br>protein 84.7 (69.7 – 102.3) g/d<br>calcium 1022.5 (816.6 – 1256.3) mg/d; phosphorus 1515.3 (1241.2 – 1823.6) mg/d; magnesium 278.2 (228.0 – 338.8) mg/d; potassium 2859.6 (2359.6 – 3436.7) mg/d; zinc 10.9 (8.8 – 13.3) mg/d	Quantitative data after log-transformation (per 100 g protein; per 100 mg minerals and 1 μg zinc)

E, energy; FFQ, food frequency questionnaire; PDS, prudent diet score; m, months; wk, week; data presented as mean values ± standard deviations or medians (interquartile range).

Table S2. Gestational age and offspring bone outcomes

Study Details					Outcome
**Country, Year [Reference]**	**Study Subjects**	**Study Design**	**Assessment Method (age)**	**Parameters**	no effect/association (↔) positive (↑) negative (↓)
UK, 2001 [31]	F=64 M=81 (neonates)	Cohort study	DXA (2 d**; 0-13 d)	WB and spine BMD	WB, spine BMD (↑)
China, 2010 [42]	F=119 M=148 (neonates)	Cross-sectional study	QUS (2.9 d*)	SOS	SOS (↑)
USA, 2003 [62]	F=50 M=45 (neonates; PT <37 wk)	Prospective study	QUS (within the first 10 d of life)	SOS	SOS (↑)
Netherlands, 2014 [10]	F=2732 M=2718 (6 y**)	Prospective cohort study	DXA (6 y**)	WB/WBLH BMD	WB/WBLH BMD (↔)
India, 2006 [24]	F=326 M=369 6.2 y**	Prospective cohort study (PMNS)	DXA (6.2 y**)	TB and total spine BMD	TB and total spine BMD (↔)
USA, 2009 [60]	M=24 6.9-7.4 y* (FT >37 wk, LP 34-37 wk, PT ≤34 wk)	Cross-sectional study	DXA, pQCT (6.9-7.4 y*)	DXA: hip, spine, FN aBMD pQCT: distal tibia cortical and trabecular vBMD	FT vs PT: hip aBMD (↑) FT vs LP: FN, hip aBMD (↑)
Turkey, 2020 [77]	F=79 M=87 (PT <37 wk– 7.5 y *; term ≥37 wk – 7.6 y*)	Prospective cross-sectional study	DXA (7.5-7.6 y*)	WB, LS, femoral BMD	PT vs term: WB BMD, LS BMD, femoral BMD (↓)

aBMD, areal bone mineral density; BMD, bone mineral density; vBMD, volumetric bone mineral density; DXA, Dual Energy X-ray Absorptiometry; FN, femoral neck; FT, full-term; LP, late preterm; LS, lumbar spine; PMNS, Pune Maternal Nutrition Study; PT, preterm; pQCT, peripheral Quantitative Computed Tomography; SOS, speed of sound; TB, total body; WB, whole body; WBLH, whole body less head; QUS, Quantitative Ultrasound; F, female; M, male; *mean, **median; wk, week (gestation); y, years

Table S3. Summary of included studies

Factor	Number of Included Studies [Reference]			
	**Total**	**Positive Effect/Association**	**Negative Effect/Association**	**No Effect/Association**
**Maternal**				
Dietary intake in pregnancy	8 [19–21,24,30–33]	7 [19–21,24,31–33]	3 [19,21,31]	1 [30]
Dietary intervention studies	7 [11-16,34]	1 [13]	0	6 [11,12,14–16,34]
Vitamin D status in pregnancy	11 [17,18,23,35–42]	5 [17,18,40–42]	0	6 [23,35-39]
Other nutrients status	6 [18,19,22-24,43]	5 [18,19,22-24]	0	1 [43]
Anthropometry	7 [24,31,52-56]	4 [31,53,55,56]	1 [53]	3 [24,52,54]
Prepregnancy weight	4 [54,57,58,59]	1 [54]	0	3 [57,58,59]
Demographic and socioeconomic factors	6 [24,52,54,60,61,62]	1 [61]	1 [54]	4 [24,52,60,62]
Smoking in pregnancy	8 [31,52,54,63–67]	0	1 [31]	7 [52,54,63–67]
**Birth-related**				
Birth anthropometry	15 [10,23,24,31,42,53,54,56,62–65,70–72]	9 [10,31,42,53,56,62,65,70,71]	0	6 [23,24,54,63,64,72]
Gestational age	7 [10,24,31,42,60,62,77]	5 [31,42,60,62,77]	0	2 [10,24]
